# Advancement in Understanding Diabetic Retinopathy: A Comprehensive Review

**DOI:** 10.7759/cureus.49211

**Published:** 2023-11-21

**Authors:** Sharad Chaurasia, Archana R Thool, Khizer K Ansari, Azeem I Saifi

**Affiliations:** 1 Medicine and Surgery, Jawaharlal Nehru Medical College, Datta Meghe Institute of Higher Education and Research, Wardha, IND; 2 Ophthalmology, Jawaharlal Nehru Medical College, Datta Meghe Institute of Higher Education and Research, Wardha, IND

**Keywords:** vascular abnormalities, ocular complications, therapeutic interventions, screening strategies, diabetic retinopathy

## Abstract

Diabetic retinopathy (DR) is a significant global health concern, with its prevalence and severity increasing alongside the rising incidence of diabetes. DR is a leading cause of vision impairment among working-age adults, resulting in substantial economic and healthcare burdens. This article explores the epidemiology and pathophysiology of DR, highlighting the global variation in its prevalence and the associated systemic risk factors. It delves into the complex relationship between glycemic control, duration of diabetes, and medication use in the context of DR development and progression. The review also discusses current screening methods and their implications, emphasizing the need for efficient and scalable approaches. Furthermore, it investigates the various treatment strategies available for DR, including laser photocoagulation, vitreous body excision, and anti-vascular endothelial growth factor (VEGF) therapy, while underlining their limitations and potential side effects. In conclusion, this article underscores the urgency of developing novel preventive and therapeutic approaches for DR. It highlights the potential role of cytokines and growth factors as treatment targets and emphasizes the importance of glycemic control and management of systemic risk factors in mitigating the impact of this vision-threatening complication of diabetes. The article serves as a comprehensive resource for understanding the challenges posed by DR and the need for innovative strategies to address this growing public health concern.

## Introduction and background

Diabetes mellitus is a set of diseases in which there is an increase and imbalance in blood glucose levels, which may be due to either impaired insulin production or systemic resistance to the effects of insulin. With > 22 million folks (7%) in the United States having diabetes mellitus, it is remarkably a health burden. Over 176 billion dollars are spent on treating diabetes economically in the United States each year, with ocular problems accounting for more than 20% of the total [1]. By 2050, it is anticipated that between a quarter and a third of all Americans will have diabetes due to an increase in its prevalence [2]. According to estimates, 5.5 million adults over 40 had diabetic retinopathy (DR) in 2005; by 2050, that figure is anticipated to rise to 16 million. In 2005, there were 1.2 million cases of vision-threatening DR; by 2050, that number will rise to 3.4 million [3].

Millions of people's everyday lives are impacted by visual difficulty, which in adults of working age is severe in industrialised nations. The majority of patients with this visual consequence were found despite rigorous glycemia, blood pressure, and lipid-lowering medication treatment by fundus examination. The count of DR patients persists in climbing, and treatment options are scarce. The present treatments come with considerable drawbacks and negative outcomes. Therefore, there is a vital necessity for the creation of novel DR prevention techniques and treatment approaches. Evidence suggests that a particular level of cytokines changes as DR develops but before clinical presentation [4].

## Review

Methodology

**Figure 1:**
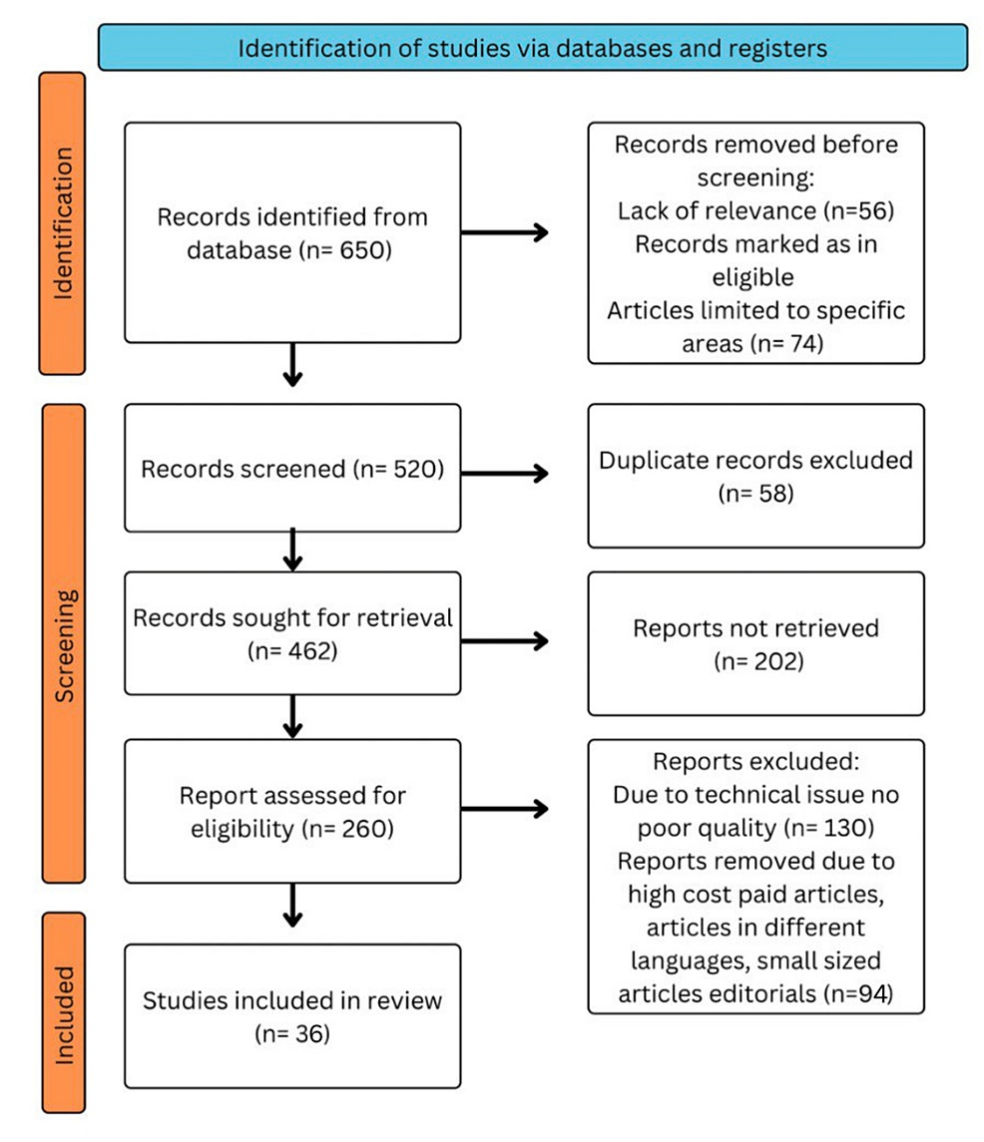
PRISMA methodology PRISMA: Preferred Reporting Items for Systematic Reviews and Meta-Analyses Image credits: Sharad Chaurasia

We conducted an extensive search across electronic databases, including PubMed, MEDLINE, Embase, Google Scholar, and ResearchGate, and explored the available English-language literature. Additionally, it was the focus of a separate inquiry. The MeSH terms were "Diabetic retinopathy" OR "Diabetes-related blindness"; "diabetic retinopathy prevalence" OR "risk factors; "development" OR "advancement"; "Pharmacological management of diabetic retinopathy" OR "Anti-VEGF therapy in diabetic retinopathy"; “Surgical management of diabetic retinopathy”; "Screening strategies for diabetic retinopathy" OR "telemedicine in diabetic retinopathy". The articles included in this review adhere to the following criteria: they encompass studies solely focused on progress in comprehending DR and novel treatment approaches, and they are studies conducted in the English language within the last two decades. Figure 1 illustrates the utilization of the Preferred Reporting Items for Systematic Reviews and Meta-Analyses (PRISMA) methodology in the research process.

Epidemiology and pathophysiology

Globally, there were 103 million cases of diabetes in 2020; by 2045, that number is predicted to rise to 161 million. The primary cause of this increase is the world's rapidly expanding diabetic population, which is concentrated in Africa, the Western Pacific, the Middle East and North Africa. There have been reports of a significant frequency of DR in North America and the Caribbean (33.30%), Middle East and North Africa (32.90%) and Africa (35.90%). The remaining areas had the following rates of DR prevalence: Western Pacific, 19.20%; South East Asia, 16.99%; South and Central America, 13.37% and Europe, 18.75% [5]. Despite the gravity of this matter, the frequency of diabetes is escalating, particularly in developing Asian nations like India (9.3%) and China (5%) by 2030 [6,7]. With rates fluctuating from 17.6% in an Indian study to 33.2% in an extensive U.S. investigation, preceding isolated studies have indicated significant variation in DR frequency estimates in individuals with diagnosed and undiagnosed diabetes [8]. Between 2005 and 2014, Taiwan had a prevalence of 0.29 to 0.35% for blindness and poor vision and 3.75 to 3.95% for diabetic eye disease [9]. From 14.3 per cent in 2006 to 15.9 per cent in 2013, DR was more common in Korea [10]. Both analyses demonstrated that females having type 2 diabetes mellitus (4%) and (16.6-17%) had a greater preponderance of DR than males (3.5%) and (12.7-14.3%), respectively. The extent of DR not only minimized well-being but also strongly anticipated death from all causes, vascular disease, and non-cancer [11].

**Figure 2:**
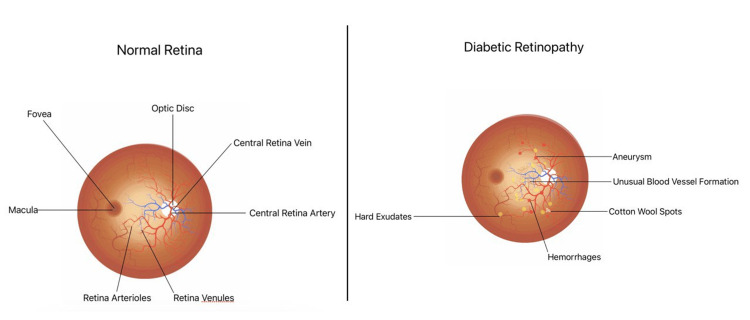
Representation of the complete retina image. The left fundus image shows a healthy retina, while the right fundus image displays the presence of diabetic retinopathy symptoms in the retina. Image credits: Sharad Chaurasia

The fact that DR is a microvascular disease has long been known. Hyperglycemia is thought to exert a major influence on the development of damage in the retinal microvasculature. A number of metabolic mechanisms have been connected to hyperglycemia-induced vascular damage, including the hexosamine route, the polyol pathway, the PKC pathway, and the accumulation of advanced glycation end products. Blood flow alterations and blood vessel dilation are the retinal blood vessels' initial reactions to hyperglycemia. In diabetic patients, these changes are regarded as a form of metabolic autoregulation designed to enhance retinal metabolism. Another feature of the initial phases of DR is the depletion of pericytes. There is evidence from both in vitro and in vivo investigations that excessive hyperglycemia causes pericytes to undergo apoptosis. Loss of pericytes causes localised capillary wall outpouching because they are necessary for capillaries' structural stability. Endothelial cell apoptosis and basement membrane thickening are also detected during the development of DR, in addition to pericyte loss, these processes jointly impair the blood-retina barrier (Figure 2) [12].

The advanced glycoxidation products are connected to the altered physiological states and the likelihood of DR, while the C-reactive protein (CRP) and homocysteine are markers of inflammation [13,14]. The plasma proteomic method has helped identify many more indicators. In DR, for instance, diphosphoinositol polyphosphohydrolase 3 alpha, CD 160 antigen, retinol-binding protein 1 (RBP1), haemoglobin subunit gamma 2 (HBG2) and neuroglobin (NGB) were downregulated and increased, respectively. The plasma level of neuroglobin, one of the five proteins listed above, shows potential utility as a diagnostic marker for DR because of the substantive variation between the groups with & without the disease. Furthermore, substantial research is being done on metabolomics, micro RNA, and genetic biomarkers [11].

Pro-angiogenic cytokines and vascular endothelial growth factor (VEGF) are thought to be the main contributors to DR neovascularization. The cytokine with profibrotic action, a connective tissue growth factor, is another potential cause of fibrosis in PDR. These are interconnected with retinal fibrosis and PDR [15]. Erythropoietin (EPO) administration and levels appeared to be most closely associated with the occurrence and severity of PDR, according to analysis by Diskin et al. [16]. A correlation between hematocrit and, most importantly, the total dose of EPO administered is suggested by the deterioration of retinopathy following the start of hemodialysis. According to another study, EPO is a strong retinal angiogenic factor that can stimulate hypoxia-driven retinal angiogenesis while functioning independently of VEGF. Inhibiting these molecular processes in retinal angiogenesis may be a fresh therapeutic approach to stop or prevent abnormal angiogenesis in DR, in accordance with study outcomes [4].

**Figure 3:**
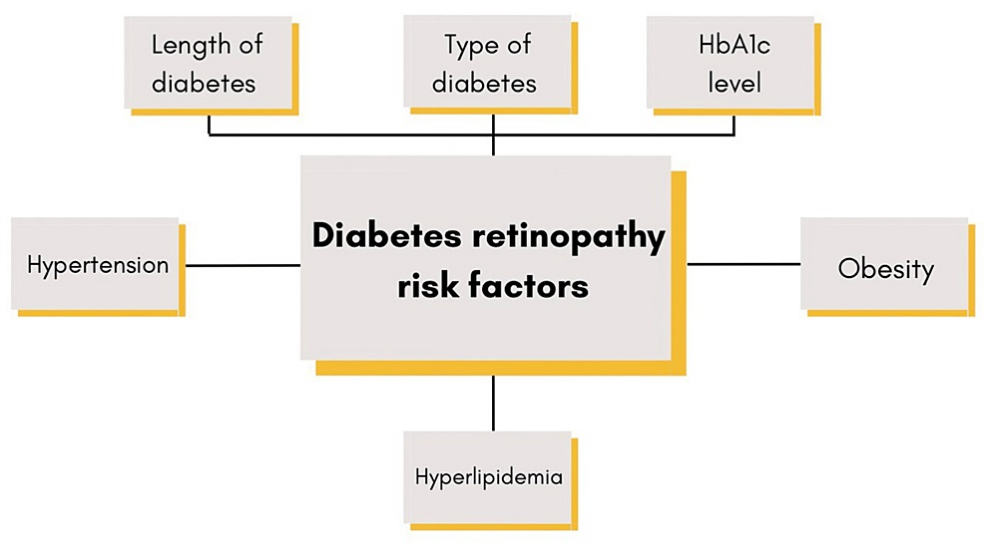
Various Diabetic retinopathy risk factors Image credits: Sharad Chaurasia

The commencement of DR is tightly connected with the type of diabetes, duration of diabetes, the level of hyperglycemia, and hypertension. Intense glycemic management lowers the incidence and worsening of DR, which is highly correlated with a higher HbA1c level [17,18]. Recent research has shown that in type 2 diabetes, DR is closely correlated with glycemic fluctuation [19]. Postprandial hyperglycemia needs to be managed in an effort to avert diabetic retinopathy. Furthermore, the link between hypertension and diabetic retinopathy is clearly demonstrated by the available research [20]. A strict blood pressure regimen slows the progression of retinopathy. Dyslipidemia, smoking, and a higher body mass index (BMI) are additional risk factors that can be changed to stop the progression of DR (Figure 3) [21,22].

Diabetes mellitus and DR

Glycemic Control

A typical measure for glycemic management monitoring is glycated haemoglobin. Numerous studies have repeatedly established that HbA1c is an autonomous risk element for DR [23]. An increased onset and advancement of DR are linked to a higher HbA1c. The LALES study discovered a one per cent increment in HbA1c and a twenty-two per cent increment in the occurrence of DR. However, the data indicated that the curve peaked at an HbA1c of about eleven per cent [24]. Elevated HbA1c is a sign of prediabetes mellitus, thalassemia, sickle cell anaemia, and uncontrolled diabetes, which is one of the prominent factors of diabetes mellitus issues, entailing DR. Even an on-target HbA1c level of seven per cent, however, was associated with an absolute risk of seven point nine per one thousand patient-years for retinal laser photocoagulation, in accordance with the United Kingdom Prospective Diabetes Study (UKPDS) [25].

Duration of Diabetes

This risk factor cannot be avoided; it has been consistently illustrated that long-term diabetes increases the odds of developing DR. According to the LALES trial, the chance of developing DR increased by 8% for every year added to diabetes history. Prolonged exposure to the hyperglycemic condition can explain this link, which may raise the risk of vascular injury and other consequences such as DR [24,25].

Drug Use in Diabetes

Individuals with DR are at a higher likelihood of needing medications to manage their diabetes, such as oral hypoglycemics or insulin. In contrast to diabetes that is undiagnosed and untreated, diabetes that is known and managed is a predictor of DR. Ninety percent of diabetics without retinopathy who participated in an exhaustive study in the Chinese population were either untreated, under diet management, or taking oral hypoglycemic medications, according to the results [26]. In contrast, oral hypoglycemic medications or insulin injections were necessary for the diabetic management of nearly 80% of individuals with DR [27]. The extent and scale of blood glucose control achieved by patients may help to explain the correlation between insulin use and DR. 

Systemic risk factors and DR

Hypertension

Time and again, studies have shown that hypertension is in conjunction with the genesis of DR. In comparison to diabetic patients who are normotensive, the Hoorn study calculated that hypertensive individuals had a prospect of retinopathy occurrence after ten years that was more than twice as high [28]. The clinical observation that diabetes and hypertension usually co-exist may help to explain this clear correlation between hypertension and DR. Hard exudates, cotton-wool patches, and retinal haemorrhages are morphological aberrations in retinal vascular system bearing similarities to those observed in mild-to-moderate NPDR and can be brought on by hypertension. Patients with DR must have their blood pressure under control, according to the influential UKPDS 69 research. The ABCD Trial authors discovered that despite having excellent blood pressure control, both groups had poor glycaemic control, which may have contributed to the advancement of DR. This would underline the significance of glycemic management in DR even more [21,24,29].

Obesity

Another risk factor that is frequently linked to cardiovascular disease is obesity. BMI, waist-hip ratio, and waist circumference can all be used to characterize it. Positive correlations exist between increased waist circumference and DR, as well as a higher waist-hip ratio [30]. Studies on the connection between BMI and DR have produced a range of conclusions. The primary risk factor for DR was shown to be obesity, defined as having a BMI of >30 kg/m^2^ in a study that primarily focused on individuals with type 1 DM, even after accounting for other risk variables, including HbA1c and the use of cardioprotective medications [30]. A separate study found that obesity and retinopathy were associated with retinopathy patients being more likely to be fat [30]. However, after adjusting for confounding factors like blood pressure, this association was no longer present. Elevated BMI value, however, was discovered to be positively correlated to deteriorating retinal vasculature condition that endangers eyesight in retinopathy patients [30].

Hyperlipidemia

Various associations between high cholesterol and DR have been unveiled in studies. A greater frequency of diabetic macular oedema and vision-jeopardizing DR was shown to be related to raised total blood cholesterol, according to Yau et al. [30]. According to the Hoorn research, there is no correlation between total cholesterol levels and the prevalence of DR. However, it did show that raised serum lipid levels are linked to a higher prevalence of the hard exudates that are characteristic of NPDR [28,30]. Elevated lipid and serum cholesterol levels are widely understood manifestations of the metabolic syndrome. Routine primary care dyslipidemia screening for diabetes patients is now feasible due to the risk variables' well-established link with events involving the cardiovascular system and mortality, additionally, the favourable impacts of medication on these risk factors are also notable. Elevated blood lipid and cholesterol levels have been linked to a prolonged-term risk of loss of vision resulting from DR. One study found that those who experienced a persistent reduction in eyesight to 5/200 or worse had an initial cholesterol score of 244 as opposed to 228 in individuals who did not [31].

Screening

Current Standards and Practices

The most up-to-date protocols and standards for the development of DR were released by the International Council of Ophthalmology in 2018 as established recommendations for diabetic eye care. In the same year, a position paper on DR was also released by the American Diabetes Association. The American Diabetes Association suggests a certain initial eye exam at a particular time based on what sort of diabetes and reasonable (level B) evidence (Table 1). A retinal examination appropriate for detecting DR and vision screening would need to be included in the standard screening test in order to ensure proper referral to an ophthalmologist.

**Table 1:** Panel: diabetes screening recommendations ADA: American Diabetes Association [32]

Type of diabetes	Time of eye examination according to ADA
Diabetes mellitus type 1	During the first five years subsequent to being diagnosed with diabetes
Diabetes mellitus type 2	Only after the diagnosis is established
Pregnant or planning to get pregnant women with prior diabetes diagnosis	Before becoming pregnant or during the first trimester, followed by monitoring during each trimester and for one year after delivery, as determined by the degree of retinopathy
Gestational diabetes	Not necessary

Opportunistic Versus Systematic Screening

Opportunistic screening happens occasionally and takes place when a patient requests a test from their physician or another healthcare provider. Not all individuals at risk may be included in opportunistic screening, and it may not be subjected to quality assurance checks. As previously said, systematic screening, on the other hand, is comprised of quality-assured preset screening procedures that involve actively identifying individuals who are at risk, keeping track of eligible subjects, and inviting them to participate in the screening programme. Every participant in the systematic screening process goes through the same screening process. The processes for invitation, selection, and follow-up are pre-established and constitute a system that provides invitations and/or notifications, as well as calls and reminders for screening at specific intervals [33].

Screening Methods

Different techniques have been used to screen for DR. This comprises wide-angle digital photography and direct ophthalmoscopy, as well as dilated stereoscopic fundoscopy, analogue fundus photography, and direct ophthalmoscopy. Many nations, including Iceland, the UK's ENSP, and France's OPHDIAT system (a telemedical network for DR screening), have implemented countrywide screening programmes. Using non-mydriatic cameras, technicians at satellite screening centres first take fundus photos for the OPHDIAT programme. After that, ophthalmologists receive these pictures via a telemedicine network for assessment. In the same manner, South India uses telescreening to detect DR in India. It entails taking 45° single-field digital fundus pictures, which are then sent digitally to retina experts for assessment. The people in the UK who grade DR go through extensive training provided by ophthalmologists, but they are not required to have medical expertise [33].

Future Perspectives of Screening

**Figure 4:**
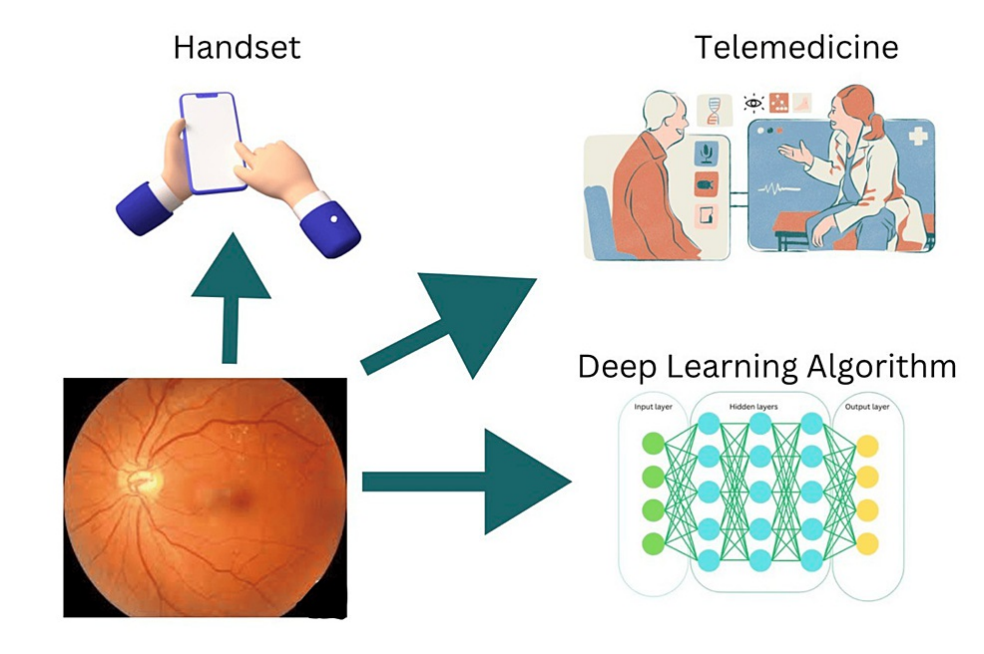
Illustration of convolutional neural network detecting diabetic retinopathy using fundus imaging and depicting ocular telemedicine Image credits: Sharad Chaurasia

Currently, skilled specialists like ophthalmologists, optometrists, or particularly trained graders screen for DR. The use of automated grading is now being investigated due to its time-consuming nature and requirement for screening big populations. In this, DR lesions are found using a computer system that processes images and recognizes patterns. The neural network method and the digital image processing technology are two different ways to recognize patterns. For recognizing and counting the initial DR lesions, such as hard exudates, haemorrhages, cotton wool patches and microaneurysms, image processing is useful (Figure 4) [33]. According to one study, DR was seen in 22.5% of the individuals in this cohort, ranging from 20% to 28%. Out of this group of patients with DR, 1.8% needed an urgent referral within 30 days because their DR was so severe, and 0.6% needed an urgent referral for non-DR-related causes. Additionally, 8.7% of people, needed ophthalmologic care within six months due to DR, and another 8.1% to 19.5% of the population needed care between 6 months and 1 year. In 23% of cases, incidental abnormalities were found throughout the screening process; most of these findings had to do with disorders such as cataracts and dry macular degeneration. In 0.6% of the screened eyes, there were incidental discoveries that were urgent or clinically significant [34]. According to one study, the cost analysis reveals that telemedicine-based DR screening is less expensive than traditional retinal examinations, which costs $49.95 and $77.80 respectively. This strategy, which makes use of digital retinal imaging and telemedicine, maybe a more practical and accessible option, especially for underprivileged and distant communities. It is imperative to encourage the broad use of telemedicine in light of the rising incidence of diabetes mellitus and DR in the US and around the world. For diabetic patients who require immediate access to eye care specialists, this can retain affordability while also greatly improving access to care and enhancing adherence to annual examinations [35].

Treatment strategies

Pharmacotherapy

As intravitreous glucocorticoids have anti-angiogenic and anti-inflammatory qualities, they are mostly used to treat diabetic macular oedema. These benefits also extend to proliferative DR, since they result in the stabilisation of the inner blood-retina barrier. Despite the absence of clinical trial data, triamcinolone acetonide is becoming widely used off-label to treat diabetic macular oedema, demonstrating its therapeutic effectiveness against the condition. Different dosages are given, varying from 4 to 25 mg, but the disadvantage is that the outcome just temporarily lasts for three months and repeated injections are required. Furthermore, about one-third of individuals might get secondary glaucoma. Dexamethasone is therefore seen as an alternate course of therapy, but with the potential adverse effect of secondary cataracts. Vascular endothelial growth factor (VEGF) plays a role in the degradation of the inner blood-retinal barrier and vascular leakage. VEGF inhibitors can inhibit the proliferation and leakage in cases of diabetic macular oedema. They are well-known for their efficacy in treating wet age-related macular degeneration. However, the duration of their impact is brief, ranging from four to six weeks. The effects of pegaptanib (an aptamer), ranibizumab (a recombinant, humanised monoclonal antibody fragment), and bevacizumab (a humanised monoclonal antibody) are now being studied in prospective multicenter studies. In individuals with diabetic macular oedema receiving treatment, randomised, double-blind research conducted in 2005 showed a substantial increase in visual acuity and a decrease in retinal thickness. There are difficulties in using ranibizumab and pegaptanib off-label because they are only approved for the treatment of wet age-related macular degeneration. Additionally, these drugs are very pricey; each injection of ranibizumab, for example, costs 1300 euros. By the end of 2011, pegaptinib and ranibizumab should be authorized for the treatment of diabetic macular oedema. In addition, research is being done on VEGF Trap Eye, a recombinant protein with an extended half-life when contrasted with ranibizumab. The USA has already approved Ozurdex, an injectable glucocorticoid with a sustained impact that lasts up to 12 months, regarding the treatment of central retinal vascular occlusion (CRVO). Additionally, investigations are underway to assess its applicability in diabetic macular oedema treatment. A range of problems, such as endophthalmitis, retinal detachment, and lens damage, are associated with intravitreous injections, sometimes referred to as intravitreous operational medication (IVOM). The complication rate is still far lower than 1%, though. IVOM operations should be carried out in aseptic operating rooms to reduce the risk of infection. In controlled research, other therapy options employing oral or injectable drugs such as somatostatin analogues (octreotide) or protein kinase C inhibitors (ruboxistaurin ) have not produced the expected outcomes. A randomised controlled trial (CALDIRET) with 635 originally recruited participants found that calcium dobesilate, when taken orally for vascular problems including venous insufficiency, could not prevent clinically significant macular oedema in people with type 2 diabetes. Only through posthoc subgroup analysis, that is, those females who had poorly managed hypertension and HbA1c levels above 9%-was a protective effect seen [36].

Laser Photocoagulation

Based on data from the prospective, randomised, controlled Early therapy Diabetic Retinopathy (ETDR) trial, which included 3,711 patients overall and was published in 1991, laser photocoagulation is the established intervention for DR and diabetic macular oedema. As a result, the Working Group on Diabetes and the Eye (Arbeitsgemeinschaft Diabetes und Auge, AGDA) and the Initiative Group for the Early Detection of Diabetic Eye Diseases (IFdA) have recommended treatments, and Germany has released national guidelines in this regard. Usually operating at a wavelength of 532 nm, the double-frequency neodymium:yttrium-aluminium-garnet (Nd:YAG) laser produces the laser utilised in this therapy. The therapy is administered through the support of contact lens applied to the cornea and a split-lamp microscope that is connected to the laser. However, Nd:YAG laser therapy might not be possible if extensive opacification of the cornea or lens obscures vision and disperses the healing light beam. In these situations, an 810 nm diode laser can be used, or the cataract can be treated initially and then a few days later, the laser can be used. When used for proliferative DR, peripheral laser photocoagulation restores the partial oxygen pressure in the non-vascular regions of the retina to normal levels, which attempts to regress newly created capillaries. Thus, there is a decreased chance of membrane development and vitreous haemorrhage. Usually, the whole surface of the retina is covered by up to 2,500 laser foci, each with a 500 μm diameter that are dispersed over the periphery but leave the centre intact. A prospective, randomised research with 1,732 eyes. The Diabetic Retinopathy Study (DRS) demonstrated that this drug decreased the risk of severe vision loss by more than 50% in 1976. 56 untreated eyes and 129 treated eyes both experienced severe vision loss. Targeted focused laser coagulation is used to close leaky microaneurysms and capillaries surrounding the fovea in cases of clinically severe diabetic macular oedema. In this instance, the laser focus sizes differed from 100 to 200 micrometers. The ETDR trial from 1985 showed a substantial reduction in the likelihood of impaired vision because of severe macular oedema. The study comprised 754 eyes that underwent targeted laser coagulation and 1,490 eyes in an untreated control group. Following rapid diagnosis and treatment, laser coagulation was promptly provided to the control group as a result of the study's findings, and this continues to be the gold standard for individuals with clinically severe macular oedema. It's crucial to remember that laser therapy seldom results in better visual acuity. Because declining visual acuity is sometimes irreversible, early diagnosis through preventative screenings is essential to maintaining acuity when the eye still has adequate vision [36].

Surgery

In the case of non-resolving vitreous haemorrhage, subhyaloid haemorrhage, tractional macular oedema, ghost-cell glaucoma and tractional retinal detachment, pars plana vitrectomy (PPV) is advised. With PPV, the retina may be repositioned, scar tissue, membranes, and hazy vitreous material can be removed. An ideal laser photocoagulation therapy can also be used. A prospective, randomised, controlled trial known as the Diabetic Retinopathy Vitrectomy trial (DRVS) demonstrated the efficacy of PPV and indicated the best time to do this treatment. Individuals who had their vitreous removed early on had far better eyesight than those who had the surgery done a year later. Recent years have seen the routine and increased efficiency of vitrectomy as a result of developments in microsurgical methods. Advancements in technology have shortened the duration of surgery and done away with the requirement for sutures. The sizes of the tools used for vitrectomy have similarly shrunk, from 1.0 to 0.6 mm. Thus, at least minimal vision is currently maintained in individuals having advanced proliferative DR. Surgical excision of the non-functioning eye may be explored as a last-ditch method of pain relief in severe situations [36].

The articles included in the review are shown in Table 2.

**Table 2:** Summary of the articles included in the review AER: Albumin excretion rate; GV: Glycemic variability; LADA: Latent autoimmune diabetes of adults; DBP: Diastolic blood pressure; DR: Diabetic retinopathy; Hcy: Homocysteine; CRP: C-reactive protein; CTGF: Connective tissue growth factor; PDR: Proliferative diabetic retinopathy; VEGF: Vascular endothelial growth factor; BMI: body mass index

Author	Year	Journal	Country	Outcomes
Gholamhossein et al., [4]	2014	Korean Journal of Ophthalmology	Iran	The risk of proliferative DR from erythropoietin was rising.
Yau et al., [8]	2012	Diabetes Care	USA	There are significant correlations between diabetic retinopathy (DR) and an extended duration of diabetes, as well as suboptimal blood pressure and glycemic control.
Song et al., [13]	2015	Plos One	USA	The level of C-reactive protein (CRP) can be employed to evaluate the extent of diabetic retinopathy (DR).
Tawfik et al., [14]	2019	Journal of Clinical Medicine	USA	Hcy may be a helpful diagnostic marker for screening diabetic individuals to determine the probabilities and severity of retinal damage.
Van Geest et al., [15]	2012	British Journal of Ophthalmology	Netherlands	A major predictor of vitreoretinal fibrosis in PDR is the CTGF/VEGF ratio.
Hainsworth et al., [17]	2019	Diabetes Care	USA	Mean HbA1c is the main modifiable risk factor for the advancement of retinopathy; additional risk variables include increased AER and DBP.
Lu et al., [19]	2018	Journal of Diabetes Investigation	China	DR and GV are more significantly correlated in type 2 diabetes than in LADA.
UK Prospective Diabetes Study Group [20]	1999	British Medical Journal	UK	Tight blood pressure control helps prevent the advancement of diabetic retinopathy and the reduction in visual acuity in individuals with hypertension and type 2 diabetes.
Chew et al., [21]	2014	Ophthalmology	USA	Intensive treatment of glycemia stops the progression of retinopathy.
Kaštelan et al., [22]	2013	Mediators of inflammation	USA	Retinopathy progresses considerably with higher BMI.
Wat et al., [30]	2016	Hong Kong Medical Journal	China	Effective control of blood pressure and glycemic levels is pivotal in mitigating the progression of diabetic retinopathy.
Vujosevic et al., [32]	2020	The Lancet	USA	By employing retinal imaging to identify individuals at risk of cardiovascular disease or cognitive impairment, the significance of diabetic retinopathy screening may be extended beyond the prevention of USA illnesses that cause visual impairment.

## Conclusions

In this comprehensive review article, the authors conducted an extensive search across multiple databases, summarizing key findings and developments in the understanding and treatment of DR over the preceding years. They covered a wide range of critical aspects related to the epidemiology, pathophysiology, systemic risk factors, screening methods, and treatment strategies for DR. The epidemiological data presented highlights the alarming rise in diabetes cases globally, with a particular concentration in regions like Africa, the Middle East, North America, and the Caribbean. The prevalence of DR varies significantly across different populations, with notable disparities between genders and types of diabetes. Furthermore, DR is not only a vision-threatening condition but also a predictor of overall health outcomes. The pathophysiology section elucidates the intricate mechanisms underlying DR, emphasizing the role of hyperglycemia in retinal microvascular damage. The review delves into various metabolic processes associated with hyperglycemia-induced vascular injury, leading to alterations in blood flow, pericyte loss, endothelial cell apoptosis, basement membrane thickening, and blood-retina barrier impairment. The article also discusses emerging biomarkers, such as advanced glycoxidation products, C-reactive protein, and neuroglobin, which offer potential diagnostic and prognostic value in DR. Pro-angiogenic cytokines and VEGF are identified as central contributors to DR neovascularization. The influence of glycemic control, duration of diabetes, drug use, hypertension, obesity, and hyperlipidemia on DR is comprehensively examined. The review underscores the significance of managing these risk factors to prevent the onset and progression of DR. The authors provide valuable insights into the current standards and practices for DR screening, comparing opportunistic and systematic screening approaches. They also discuss the various screening methods, including telemedicine-based solutions, highlighting their cost-effectiveness and potential to improve access to care. The treatment strategies section explores pharmacotherapy, laser photocoagulation, and surgery as viable options for managing DR. Intravitreous glucocorticoids and anti-VEGF agents are discussed as key interventions for diabetic macular oedema, while laser photocoagulation remains the gold standard for the treatment of DR. Pars plana vitrectomy is considered for cases of non-resolving vitreous haemorrhage and retinal detachment. In conclusion, this comprehensive review article provides a thorough overview of the current state of knowledge regarding DR. It emphasizes the importance of early screening, risk factor management, and advances in treatment modalities. The article underscores the need for ongoing research and innovation to address the growing global burden of DR and its significant impact on public health.
